# Community health workers: A crucial role in newborn health care and survival

**DOI:** 10.7189/jogh.04.020302

**Published:** 2014-12

**Authors:** Samira Aboubaker, Shamim Qazi, Cathy Wolfheim, Adebowale Oyegoke, Rajiv Bahl

**Affiliations:** 1 World Health Organization; 2 King's College London School of Medicine

There is ample evidence from research and implementation to show that community health workers (CHWs), when appropriately trained, supplied, supported and supervised, can identify and correctly treat most children for pneumonia, diarrhoea and malaria [1,2]. Community management of childhood illness is an important contribution to the remarkable progress in reducing child mortality. Globally, the rate of under–five mortality has decreased by nearly half, from 90 deaths per 1000 live births in 1990 to 46 in 2013 [3].

However, in the same time period the decrease in newborn deaths has been significantly lower. The neonatal mortality rate has seen an annual decrease of only 2%, falling from 33 deaths per 1000 live births in 1990 to 20 in 2013. In consequence, the proportion of under–five mortality taking place in the first month of life has increased.

The 2.8 million newborns who died in 2013 represent 44% of all under–five mortality. In addition, most of these deaths took place during the first 24 hours after birth, and were due to conditions that can be prevented or treated with effective, existing interventions: prematurity, birth asphyxia and neonatal infections. The first 24 hours are also considered the most dangerous time period for a new mother [4].

This paper shows how a programme to reduce newborn mortality through the training and deployment of CHWs can lead to significant improvements in survival rates of newborns and mothers.

A fundamental principle underpinning the delivery of effective maternal, newborn and child health interventions is the continuum of care. This continuum involves the seamless provision of care during pregnancy, delivery, as well as the newborn and infant periods. It encompasses home care, visits to the health facility/hospital, and follow–up in the community. The continuum of care is a cornerstone of the UN Secretary–General’s Global Strategy for Women’s and Children’s Health [5], and is reflected in the Every Newborn Action Plan launched in June 2014 [6].

Community health workers play a vital role in facilitating that continuum of care, acting as the bridge between the community and the health facility. WHO and UNICEF have produced a set of materials titled *Caring for Newborns and Children in the Community.* This set comprises three packages for training and supporting CHWs. These are, in brief:

*Caring for the Newborn at Home*: The CHW counsels women during five home visits: two during pregnancy; one on the day of birth if the mother gave birth at home, or soon after she has returned home from the health facility; and on days 3 and 7 after birth. Additional visits are proposed for low birth weight babies.

*Caring for the Child’s Healthy Growth and Development*: The CHW counsels families on practices that they can carry out at home, concerning infant feeding; communication and play for child development; recognition of and response to childhood illness; and illness prevention (handwashing, vaccination, use of bednets).

*Caring for the Sick Child in the Community*: The CHW assesses, classifies and treats children aged 2 months to 5 years with pneumonia, diarrhoea and/or malaria, and assesses for malnutrition. The treatment interventions include the use of four simple medicines: an antibiotic, an antimalarial, oral rehydration salts (ORS) and zinc tablets.

Most programmatic experience to date has been with caring for the sick child in the community, also known as integrated community case management, or iCCM. This curative care is indisputably important, and implementation is expanding in many countries. At the same time, there are many other tasks that CHWs can effectively carry out. The experience in iCCM can inform decision–makers to review the best means of using CHWs in strategies for improving newborn survival.

## HOME VISITS FOR THE CONTINUUM OF CARE

Strategies to train and deploy community health workers show great promise in increasing access to treatment and care of pregnant women and their newborns. While the jury is still out on the programmatic feasibility of CHW treatment of sick newborns, there is ample evidence on the value of home visits during pregnancy and after birth to promote maternal and newborn survival. Home visitation is an effective strategy to deliver care, improve key newborn care practices, and identify signs of maternal and newborn illness, especially in settings of high neonatal mortality. The UNICEF/WHO Joint Statement Home Visits for Newborn Care [7] puts forward a series of recommendations and describes the evidence behind each of them, while taking into account programmatic considerations.

**During pregnancy:** According to the 2014 report Fulfilling the Health Agenda for Women and Children [8], nearly half of pregnant women in developing countries still do not attend antenatal care visits, and 37% receive no skilled care during delivery. CHWs can play a fundamental role in advising, encouraging and empowering families to seek antenatal care from a qualified health worker. They also help the family to prepare for delivery by ensuring they know where to go and helping them overcome barriers concerning money, transportation, and other necessary family logistics.

A CHW trained in the appropriate WHO/UNICEF package identifies pregnant women in the community, and makes two home visits to each one [9].

**Postnatal care:** The days and weeks following childbirth – the postnatal period – is a critical phase in the lives of mothers and newborn babies. Events occurring during this period can have a major effect on the well–being of a mother and her newborn.

It is also the most vulnerable time, when most maternal and infant deaths occur. It is nonetheless the most neglected period for the provision of quality services. The recently–published document “WHO Recommendations on Postnatal care of the Mother and Newborn” [10] summarizes the evidence for a series of recommendations that address the timing, number and place of postnatal contacts, as well as the content of postnatal care for all mothers and babies during the six weeks after birth. The recommendations also include assessment of mothers and newborns to detect problems or complications. CHWs can provide a number of these postnatal care services and interventions for lactating women and their newborns.

**Pre–and post–natal visits:** Studies conducted in Bangladesh, India and Pakistan [11–15] have shown that home visits can reduce newborn deaths in high–mortality, developing country settings by 30% to 61%. The visits also improved coverage of key newborn care practices in the home. This evidence complements the experience from developed country settings showing that postnatal home visits are effective in improving breastfeeding rates and parenting skills [16].

An additional observational cohort study in Bangladesh showed the effect of postnatal care home visits by trained CHWs on neonatal mortality rates [17]. The study showed that among infants who survived the first day of life, neonatal mortality was 67% lower in those who received a visit on day one than in those who received no visit. For those who survived the first two days of life, receiving the first visit day two was associated with 64% lower neonatal mortality than those who did not receive a visit.

A cluster–randomized controlled trial in the United Republic of Tanzania investigated the effect of home–based counselling on newborn care practices [18]. In this study, trained volunteers made home visits during pregnancy and after childbirth to promote recommended newborn care practices including hygiene and breastfeeding. They also identified and provided extra care for low birth weight babies. Improvements in home care practices included delaying the baby’s first bath by at least six hours (81% in intervention areas vs 68% in control areas), exclusive breastfeeding in the three days after birth (83% vs 71%), putting nothing on the cord (87% vs 70%), and, for home births, tying the cord with a clean thread (69% vs 39%).

**Figure:**
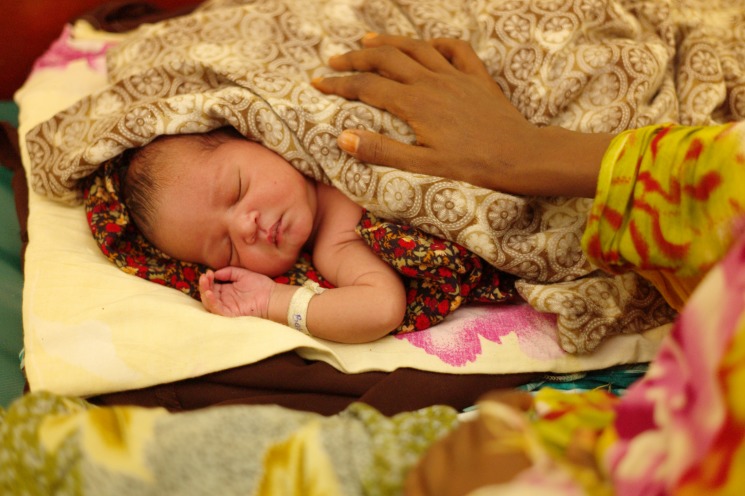
Photo: Courtesy of Christine Nesbitt, UNICEF (©UNICEF/CHRISTINE NESBITT)

A systematic review of randomized controlled trials [19] looked at the effect of home–based newborn care provided by community health workers on preventing neonatal, infant and perinatal mortality. The trials, all from South Asian countries, included home visits during pregnancy (4 trials), home–based preventive and/or curative neonatal care (all trials), and community mobilization efforts (4 trials). The intervention was associated with a reduced risk of mortality during the neonatal and perinatal periods. In one trial, a significant decline in infant mortality was documented. Subgroup and meta–regression analyses suggested a greater effect with a higher baseline neonatal mortality rate. This review further strengthened the argument for home–based neonatal care strategies in South Asian settings with high neonatal mortality rates and poor access to health facility–based care.

The Newhints cluster–randomized trial in Ghana showed the effects of home visits by trained community–based surveillance volunteers (CBSVs) on neonatal mortality and home care practices [20]. The CBSVs in the study zones were trained to identify pregnant women in their community and to make two home visits during pregnancy and three in the first week of life. During these visits they promoted essential newborn care practices, weighed and assessed babies for danger signs, and referred as necessary. The intervention achieved a reduction of 8% in the overall neonatal mortality rate (NMR), which are less than the results observed in South Asia. The meta–analysis summary estimate is a 12% reduction in NMR.

In an evaluation of a cluster–randomized controlled trial of a package of community–based maternal and newborn interventions in Bangladesh [13], CHWs identified pregnant women; made two antenatal home visits to promote birth and newborn care preparedness; made four postnatal home visits to negotiate preventive care practices and to assess newborns for illness; referred sick neonates to a hospital and facilitated compliance in the intervention sites. The study led to high coverage of antenatal home visits (91% visited twice) and postnatal home visits (69% visited on days 0 or 1). Although there was no impact on neonatal mortality, there were clear improvements in newborn care practices and care seeking.

A 2008 pilot study by Bhutta et al [21] investigated the feasibility of delivering a package of community–based interventions for improving perinatal care using lady health workers (LHWs) and traditional birth attendants (Dais) in rural Pakistan. The LHWs were trained on essential maternal and newborn care. They conducted community education group sessions, were encouraged to link up with local Dais and were supported by voluntary health committees. The interventions led to a reduction in neonatal mortality (from 57.3 to 41.3 per 1000 live births), and an increase from 18% to 30% in the proportion of facility deliveries. There was also an increase in key newborn care practices such as early and exclusive breastfeeding, delayed bathing and cord care.

Recent data available from the AFRIcan Neonatal Sepsis Trial (AFRINEST) studies in the Democratic Republic of the Congo, Kenya and Nigeria [22,23], show that CHWs can adequately assess a newborn for signs of illness, weigh the infant, and refer for medical care if needed.

In Ethiopia, Health Extension Workers make home visits and also manage sick newborns in the community. It should be noted that these are not typical CHWs, as they benefit from almost a full year of pre–service training as well as a national policy that allows them to treat newborns with infection.

## CONCLUSION

Accelerated action against the main child killers is imperative as countries work to reduce the under–five mortality rate and achieve the fourth Millennium Development Goal by 2015. The adequate reduction of under–five mortality requires increased attention to newborns, and in the landscape of the continuum of care, to women before and after giving birth. This must be done by reaching out to underserved populations to provide them with the essential health services they need.

There is widespread consensus on the central role that community health workers can play in ending preventable maternal, newborn and child deaths. The Every Newborn Action Plan sets out a clear vision. Policy and recommendation documents provide the most up–to–date information, and training materials are available to support the implementation of a community–based strategy.

CHWs can identify pregnant women and newborns in need of medical attention and care, promote and encourage appropriate careseeking, and provide counselling and support for home care practices across the periods of pregnancy, newborn and childhood. As the fundamental link between a community and its health facility, and between the population and the health workers, CHWs can also promote adherence to treatment and follow–up. Community health workers are an important option for investment as part of a comprehensive primary health care system. Effective implementation of CHW strategies requires policy support, training, supervision, performance maintenance and regular supplies. In addition, community health workers are increasingly responsible for many health and development tasks, and expansion of their duties needs to be carefully considered in this light.
